# A NEW FEATURE OF JAPANESE CAREGIVING? COMPOUND CAREGIVING OF OLDER ADULTS IN AN AGING SOCIETY WITH FEWER CHILDREN

**DOI:** 10.1093/geroni/igz038.2243

**Published:** 2019-11-08

**Authors:** Tomoko Wakui, Tomoko Wakui, Emily M Agree, Ichiro Kai

**Affiliations:** 1 Tokyo Metropolitan Institute of Gerontology, Tokyo, Japan; 2 Tokyo Metropolitan Institute of Gerontology, Itabashiku, Tokyo, Japan; 3 Johns Hopkins University, Baltimiore, Maryland, United States; 4 The University of Tokyo, Bunkyoku, Tokyo, Japan

## Abstract

Due to the combined effects of longevity of aging parents, fewer children, and caregiving traditions, family members face multiple caregiving responsibilities in Japan. This study examined the emergence of compound caregiving--providing care to multiple adults-- and the relationship of caregiving status to burden, depression, and social support. Data were from the Fukui Longitudinal Caregiver Study, a survey of family caregivers to older Japanese adults who received long-term care services. We analyzed data from 2,025 caregivers whose mean age was 63 years old. Results showed that 9.5% of caregivers provided care for more than two care recipients. Compared to single caregivers, compound caregivers were more significantly burdened. Compound caregivers who reported higher instrumental and informational support from live-in family and higher emotional support from friends showed significantly lower caregiving burden. We discuss how traditional caregiving norms and demographic changes lead to new needs for family support in Japan’s aging society.

